# The characterization of surgical smoke from various tissues and its implications for occupational safety

**DOI:** 10.1371/journal.pone.0195274

**Published:** 2018-04-12

**Authors:** Markus Karjalainen, Anton Kontunen, Sampo Saari, Topi Rönkkö, Jukka Lekkala, Antti Roine, Niku Oksala

**Affiliations:** 1 BioMediTech Institute and Faculty of Biomedical Sciences and Engineering, Tampere University of Technology, Tampere, Finland; 2 Aerosol Physics, Faculty of Natural Sciences, Tampere University of Technology, Tampere, Finland; 3 Department of Surgery, Hatanpää Hospital, Tampere, Tampere, Finland; 4 Division of Vascular Surgery, Tampere University Hospital and Faculty of Medicine and Life Sciences, University of Tampere, Tampere, Finland; CHU Clermont-Ferrand, FRANCE

## Abstract

Electrosurgery produces surgical smoke. Different tissues produce different quantities and types of smoke, so we studied the particle characteristics of this surgical smoke in order to analyze the implications for the occupational health of the operation room personnel. We estimated the deposition of particulate matter (PM) from surgical smoke on the respiratory tract of operation room personnel using clinically relevant tissues from Finnish landrace porcine tissues including skeletal muscle, liver, subcutaneous fat, renal pelvis, renal cortex, lung, bronchus, cerebral gray and white matter, and skin. In order to standardize the electrosurgical cuts and smoke concentrations, we built a customized computer-controlled platform. The smoke particles were analyzed with an electrical low pressure impactor (ELPI), which measures the concentration and aerodynamic size distribution of particles with a diameter between 7 nm and 10 μm. There were significant differences in the mass concentration and size distribution of the surgical smoke particles depending on the electrocauterized tissue. Of the various tissues tested, liver yielded the highest number of particles. In order to better estimate the health hazard, we propose that the tissues can be divided into three distinct classes according to their surgical smoke production: 1) high-PM tissue for liver; 2) medium-PM tissues for renal cortex, renal pelvis, and skeletal muscle; and 3) low-PM tissues for skin, gray matter, white matter, bronchus, and subcutaneous fat.

## Introduction

Electrosurgery is an essential tool in a surgeon’s repertoire and is now used in almost every surgical procedure. It is used both to cut tissue, and to control bleeding by coagulating the blood vessels. The procedure involves administering a high-frequency electric current through the target tissue, causing its temperature to increase [1]. The heating effect of the surgical instrument used for the procedure is controlled by the waveform of the current. A low-voltage, high-frequency current causes a rapid increase in temperature, causing the tissue to evaporate rapidly; essentially cutting the tissue. A high-voltage, low-frequency current results in a more gradual heating effect that denatures the proteins in the tissue, resulting in the coagulation and occlusion of the affected blood vessels [2]. In modern electrosurgical procedures, high-frequency “cut” and low-frequency “coag” modes are interweaved to achieve a clinically optimal combination of cutting and coagulation. It is the evaporation of the tissue that produces the plume of smoke, herein referred to as “surgical smoke” (SS).

SS causes technical, physical, and occupational health problems. One obvious challenge is the visual obfuscation that occurs, particularly in laparoscopic surgery. SS has also been shown to contain living bacteria [3] and viruses [4], thus exposing surgical staff to the risk of infection. In addition, exposure to aerosol particles is associated with an increased risk of respiratory diseases and strokes [5–10]. Some of the harmful effects are mediated by carcinogenic volatile molecules such as acrylonitrile (a precursor of cyanide) and carbon monoxide [11]. Other health hazards are caused by airborne particles, especially those with a diameter at or below 2.5 μm (PM_2.5_), as these are known to have long-term negative health effects. Such particles are able to penetrate the defense mechanisms of the upper respiratory tract and enter the alveoli, as well as systemic circulation. The deposition of inhaled particles has been documented in the brain, liver, heart and kidneys [12–14]. The standard surgical mask alone does not protect the wearer from SS due to leakages around the mask and the filtration efficiency of small particles [15,16]. Even though the risks of SS are acknowledged, smoke evacuation units are not yet routinely used in many healthcare centers [17,18].

There has been little research in the literature on the effects exposure to SS has on operation room personnel during various clinical procedures. Although one study [18] has provided evidence of particle production during a specific surgical procedure, the procedures followed for electrosurgery vary from surgeon to surgeon. To our knowledge, there is no data available on the composition and levels of the particles in SS from different tissues with standardized electrosurgery. Therefore, our goal was to analyze the particle composition of SS from various types of landrace porcine tissues during standard electrosurgical procedures in order to estimate the potential lung depositions of these particles, and their implications for the health of the surgical personnel in the operating room.

## Materials and methods

### Particle analyzer

We used an electrical low pressure impactor (ELPI, Dekati Inc., Finland) [19] with a filter stage [20] and an additional impactor stage [21] for real-time particle number, mass, and size distribution measurements. The ELPI measures the concentration of all the aerodynamic particles with a diameter between 7 nm and 10 μm. We calculated the particle mass distribution from the ELPI number distribution using standard water density for the particles (1 g/cm^3^). This approximation in particle mass calculations from ELPI data typically gives correct results [22]. Because of the high particle concentration, we first diluted the smoke sample using an ejector type diluter (Dekati Diluter DI-1000, Dekati Inc.) with a 1:8 dilution ratio, and a second dilution step was performed by mixing 8.5 l/min pure air with 2 l/min sample flow from the Dekati Diluter. Therefore, the total dilution ratio was 1:45.

### Testing platform

**Fig 1:**
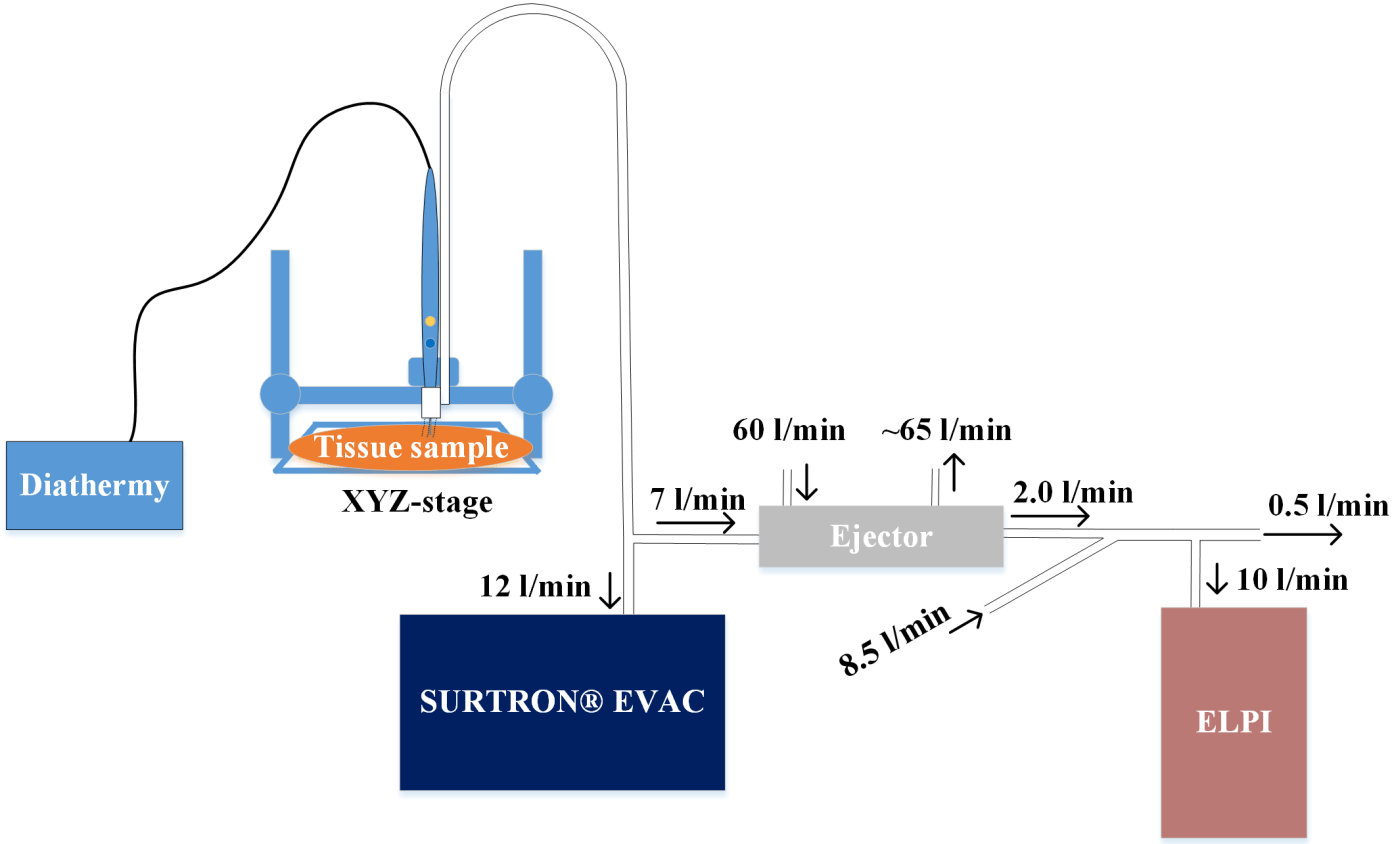
The measurement system.

In order to control the smoke production, we standardized the electrosurgical cuts (i.e. length, depth and duration of the cut) with a custom-made computer-controlled platform, a schematic diagram of which is presented in Fig 1. In the system, a diathermy knife was moved with an automated xyz-stage and the measured tissue sample was placed on a custom-built ground electrode. We used conductive tubes (Tygon, Saint-Gobain, France) for aerosol sampling to minimize the electrical losses of the particles. The tubes were connected to a commercially available smoke evacuator (Surtron Evac, Quirumed, Spain). The evacuator power was set at five (out of nine), which equals a suction of 12 l/min. We measured the flow settings with a commercial flow calibrator (Gilian Gilibrator 2, Sensidyne, Germany). We collected the smoke at 2 cm from the diathermy tip, so that a high proportion of the smoke was captured. We conducted the measurements in a laboratory located in the faculty of Biomedical Sciences and Engineering at Tampere University of Technology. During the study, the testing platform was located inside a fume hood in order to prevent the measurement laboratory being contaminated by the smoke.

### Testing platform evaluation with a weight scale

We tested tissue weight loss during diathermal cutting with a regular weighing scales (XR 205SM-DR, Precisa, Switzerland). We used a cutting time of 7 seconds, and the mean weight loss of liver during the nine tests was 41 (±12) mg, which suggests an evaporation speed of 5.9 (±1.2) mm^3^/s, assuming a unit mass of 1 g/cm^3^. In a surgical evacuator stream of 12 l/min, this would indicate that there could be a maximum mass concentration of 29 g/m^3^ of aerosol particles, discounting any losses. According to the ELPI measurements, the calculated total mass concentration of aerosol particles from the liver was 9.1 (±4.2) g/m^3^, which would indicate a loss of 20 (±9.2) g/m^3^. This indicates significant losses from the evaporated tissue mass into the measured aerosol mass. The diathermy knife and the head of the suction tube collected a noticeable amount of evaporated tissue matter, indicating that some of the evaporated mass is deposited near the surgical event. Additional losses in concentration, which are not detectable in the ELPI measurements, could be due to the particulate mass vaporizing into gaseous molecules. The diathermy smoke may contain up to 95% water [23], although in our experiment the amount of particulate-gas in the evaporated water was undefined.

### Diathermy device

We used a commercially available electrosurgery unit (Itkacut 350MB, Innokas Medical, Finland) set at a nominal 120 W power. Such a high power level was used to avoid the sample sticking to the diathermy tip. We used direct cut settings at a cutting frequency of 450 kHz. We made a flat, steel custom-ground electrode (19 x 19 cm) for the robot stage, and measured the cutting voltage at slightly under 800 V peak-to-peak.

### Samples

**Fig 2:**
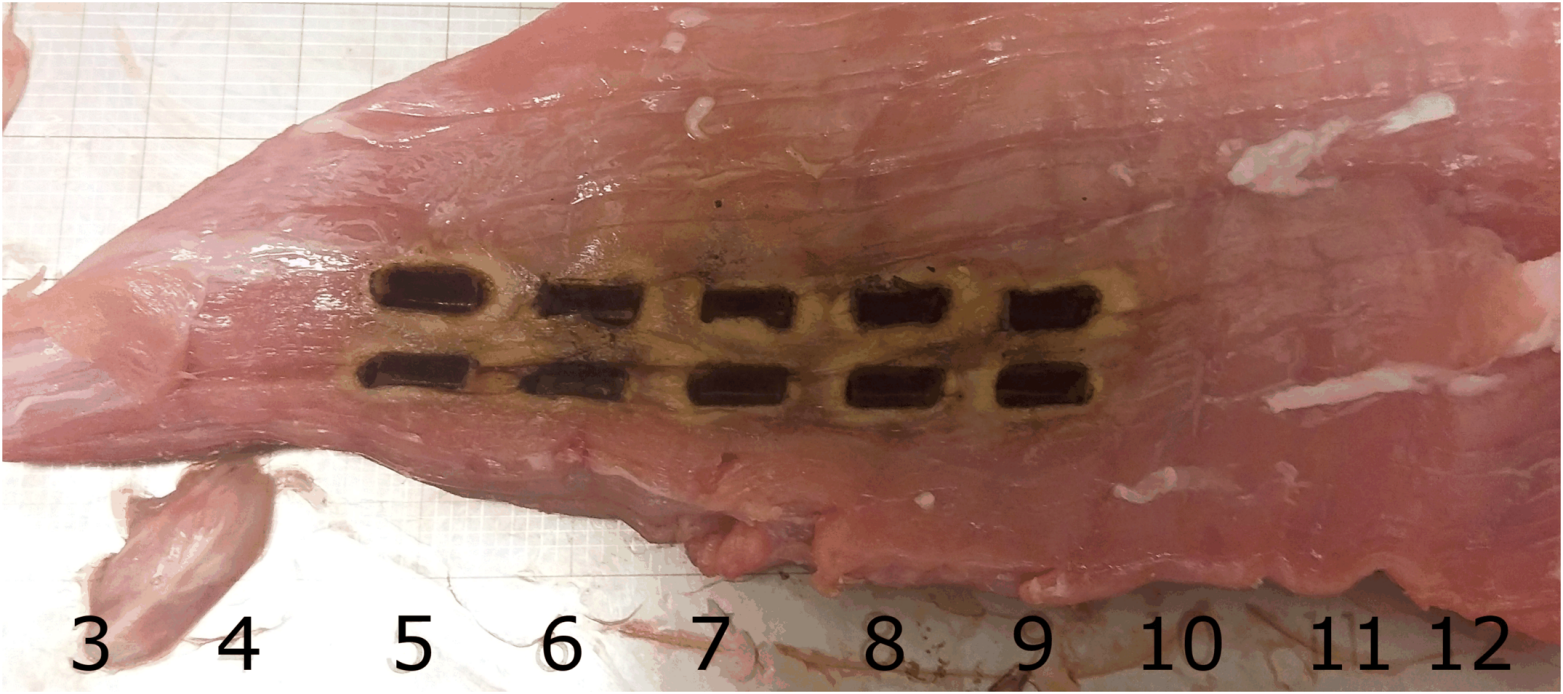
Representative image of a pig skeletal muscle sample after being exposed to the ten sample burns.

The test materials were fresh, (unfrozen) Finnish landrace porcine tissues, purchased from a local slaughterhouse (Paijan Tilateurastamo, Urjala, Finland). The smoke from ten different tissue types was measured: skeletal muscle, liver, subcutaneous fat, renal pelvis, renal cortex, lung, bronchus, cerebral gray and white matter, and skin, all taken from the same animal. We performed the sampling with the automated xyz-stage to ensure that the cuts were all the same size. A typical cutting pattern is presented in Fig 2. The numbers and dark lines in the Fig 2 show the 10 mm spacing in the ground electrode. Each electrosurgical cut was 5 mm in length, and we took ten test samples from every tissue type. The 2.4 mm-wide blade (HF 9805–24 Hebu medical, Germany) had a sharp tip, and we aimed for 4 mm deep-cuts, although these varied slightly due to variations in the height of the tissue.

### Particle deposition model

Only a certain proportion of the emitted particles will be deposited in the respiratory tract, and so we calculated the level of deposition with models used by the International Commission on Radiological Protection (ICRP, 1994) [15]. We estimated the airway deposition fractions for the upper airways (UA), the bronchial tube (BT) and the alveoli (AL) from the measured aerosol particle mass distributions. We calculated the particulate matter (PM) depositions from these distributions using a unit density of 1 g/cm^3^. The toxicity of atmospheric aerosols is commonly calculated from the PM values, which are typically PM_10_, PM_2.5_, and PM_1_ for particulate total mass for particles with diameters smaller than 10 μm, 2.5 μm, and 1 μm, respectively.

The sample size was relatively small for statistical evaluation of the data, having only ten particle measurements per tissue. This small sample size was necessary in order to maintain the heterogeneity of the tissues, which limited their physical size. For example, although we had plenty of excess liver tissue, which would have been enough for several additional measurements, there was only enough macroscopically homogenous tissue area from certain tissues, such as the gray and white matter, for ten electrosurgical cuts if all the samples were to be taken from a single animal.

We performed a two-sample analysis with a confidence interval (CI) of 99% between the mass distributions of every different tissue. We subjected the sample groups to the Shapiro-Wilk normality test individually. The test indicated non-normality within the sample groups and thus a non-parametric method was chosen to validate the statistical difference between the levels of various tissue SS. Since all the tissues came from the same animal, the Mann-Whitney U test was used for the analysis.

### An inverse spherical model

We evaluated the SS hazard for OR personnel with an inverse spherical model [24], in which the smoke intensity decreases inversely to the square of the distance from the source. Since we used an unusually high power for the surgical diathermy (120 W), we made a linear assumption for the smoke that would be produced with 40 W, which is the most common power limit in surgical operations. This linear assumption is in line with previous results in the literature [25]. In our model, we also used smoke evacuation efficiencies reported in the literature for integrated and general-purpose evacuation. In an integrated smoke evacuator, the smoke suction is integrated with the electric scalpel, which produces a high suction efficiency of 88% [26]. A general purpose surgical suction is commonly a hand-help pump unit, used for collecting blood and other liquids, which is not optimized to remove SS. The smoke suction efficiency of the general purpose evacuation is approximately 50% [24].

## Results

**Fig 3:**
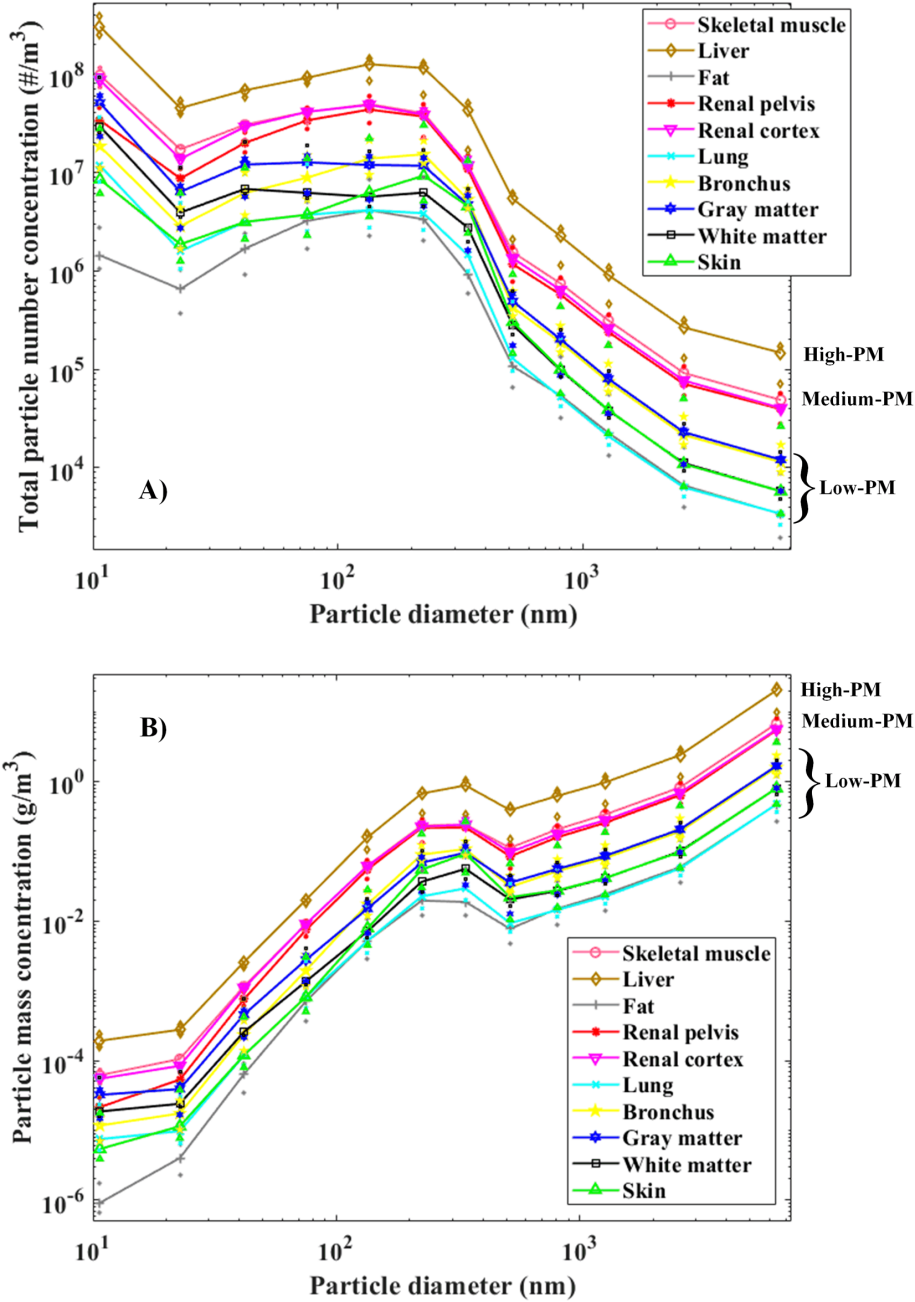
Median particle number (A) and mass (B) concentrations of different tissue types, produced with the diathermy knife. The particulate concentrations form three groups: high-PM, medium-PM, and low-PM.

The particulate number distributions of each tissue type are presented in Fig 3A, while Fig 3B shows the mass distributions. The curves in the Fig 3 are the medians from ten tests. The results of each individual test can be found in S1 Dataset and S1 File. Based on the corresponding particle number and mass distributions (Fig 3), three tissue type groups can be distinguished: high-PM tissues, medium-PM tissues, and low-PM tissues. The mass distributions varied significantly between the tissue types and the particle sizes, so logarithmic distribution axes were used. Furthermore, due to the diffusional losses of the smallest particles in the ELPI stages, the accuracy of the presented PM values increases as a function of decreasing particle size.

**Fig 4:**
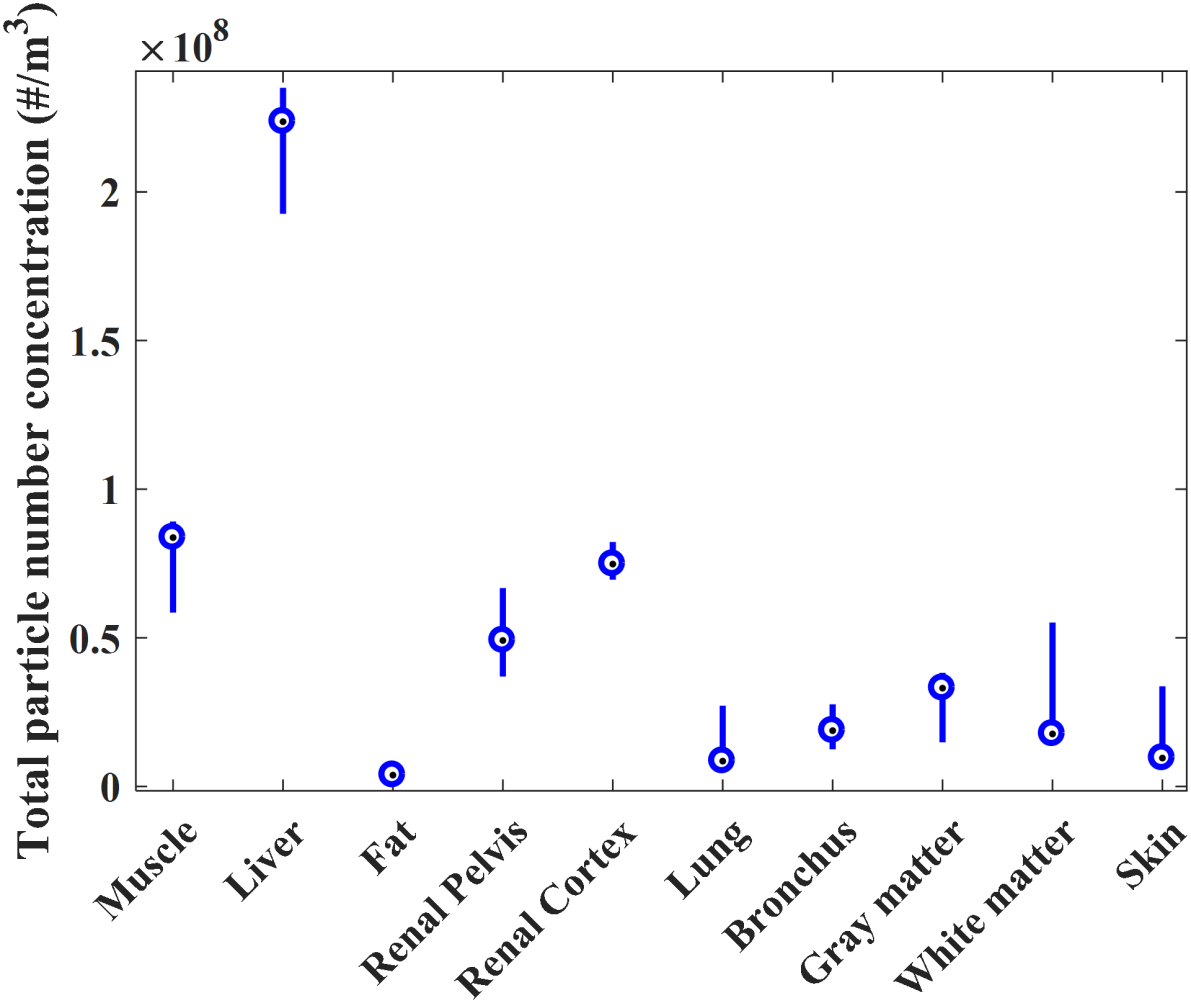
Boxplot presentation of the distributions of the measured total particle number for each tissue. Medians are presented as dots between the quartile lines.

The majority of mass in the smoke is explained by the large particles, as can be seen from the curve trends in Fig 3B. In contrast, the particle number concentration is highest in the smallest particle size range (Fig 3A). The particle size distribution curves presented in Fig 3 indicate that there are at least two particle modes: the first around 10 nm and the second around 100 nm. The variations in the concentration of the total number of particles between the individual tests are presented in Fig 4. The variations within each tissue, and the deviation of the liver from the other tissues, are clearly visible. Some of the tissues, such as those of the renal cortex and bronchus, only exhibited moderate variation (<1.3x10^7^ #/m3 difference in the particle number concentration between the first and third quartiles), but the variation between the quartiles in the liver tissue was substantial (>4.2x10^7^ #/m3) (Fig 4).

The deposition fractions of the different mass fractions, PM_10_, PM_2.5_ and PM_1_, are presented in Table 1, which shows that PM_2.5_ and PM_1_ particles constitute only slightly more than one tenth of the total particulate mass.

**Table 1:** Measured aerosol median masses inside the surgical smoke evacuation stream from different tissue types, and the calculated mass depositions to the upper airways (UA), the bronchus (B), and the alveoli (AL) for particles under 10 μm (PM_10_), under 2.5 μm (PM_2.5_) and under 1 μm (PM_1_).

**PM**_**10**_ **(mg/m**^**3**^**)**	**Muscle**	**Liver**	**Fat**	**Renal pelvis**	**Renal cortex**	**Lung**	**Bronchus**	**Gray matter**	**White matter**	**Skin**
Measured total mass	3000	9100	210	2400	2500	210	720	760	370	370
Mass deposited to UA	1900	5700	130	1500	1600	130	440	470	230	220
Mass deposited to B	110	320	7.4	85	88	7.4	25	26	13	13
Mass deposited to AL	160	460	11	120	130	11	37	39	19	20
**PM**_**2.5**_ **(mg/m**^**3**^**)**	**Muscle**	**Liver**	**Fat**	**Renal pelvis**	**Renal cortex**	**Lung**	**Bronchus**	**Gray matter**	**White matter**	**Skin**
Measured total mass	370	1100	28	300	330	29	100	100	52	61
Mass deposited to UA	110	320	7.9	85	92	7.7	26	28	14	14
Mass deposited to B	23	66	1.6	18	19	1.6	5.4	5.8	2.8	2.8
Mass deposited to AL	53	160	3.9	42	46	3.9	14	14	7.0	7.4
**PM**_**1**_ **(mg/m**^**3**^**)**	**Muscle**	**Liver**	**Fat**	**Renal pelvis**	**Renal cortex**	**Lung**	**Bronchus**	**Gray matter**	**White matter**	**Skin**
Measured total mass	150	470	12	130	140	14	52	47	25	34
Mass deposited to UA	9.9	31.0	0.74	8.0	8.9	0.83	3.0	3.0	1.6	1.9
Mass deposited to B	1.6	4.9	0.12	1.4	1.5	0.14	0.49	0.48	0.25	0.29
Mass deposited to AL	12	38	0.94	10	11	1.1	3.9	3.7	2.0	2.5

A statistical analysis of the total particle masses revealed that only the liver was significantly different from the rest of the tissues. The results for all the tissues can be seen in Table 2, as the p-values of the two-sample Mann Whitney U test (99% CI). Any p-values under 0.01 are in bold type.

**Table 2:** The result matrix for the p-values of the statistical analysis* based on the total mass of the particles created in tissue electrosurgery.

	**Muscle**	**Liver**	**Fat**	**Renal pelvis**	**Renal cortex**	**Lung**	**Bronchus**	**Gray matter**	**White matter**	**Skin**
**Muscle**		**0.0028**	**0.0006**	0.5205	0.1041	**0.0002**	**0.0002**	**0.0002**	**0.0002**	**0.0003**
**Liver**	-		**0.0003**	**0.0028**	**0.0028**	**0.0002**	**0.0002**	**0.0002**	**0.0002**	**0.0004**
**Fat**	-	-		**0.0022**	**0.0017**	0.9698	0.0452	0.1859	0.1212	0.3847
**Renal pelvis**	-	-	-		1.0000	**0.0002**	**0.0006**	**0.0002**	**0.0006**	**0.0028**
**Renal cortex**	-	-	-	-		**0.0002**	**0.0002**	**0.0002**	**0.0002**	**0.0002**
**Lung**	-	-	-	-	-		0.0257	0.1859	0.0757	0.3447
**Bronchus**	-	-	-	-	-	-		0.4727	0.2123	0.3847
**Gray matter**	-	-	-	-	-	-	-		0.9698	0.6776
**White matter**	-	-	-	-	-	-	-	-		0.7913
**Skin**	-	-	-	-	-	-	-	-	-	

*Mann-Whitney U test (99% CI).

We concluded from both Tables 1 and 2 that even though some of the tissues are similar in terms of the mass of the produced particles, three separate groups can be distinguished, based on their corresponding particle mass size distributions, as seen in Fig 3, where three PM-classes are presented.

Table 3 shows estimates of the particle concentrations from various distances. Three user cases are included: surgery without any smoke removal, surgery with a general-purpose suction device and surgery with an integrated smoke evacuator. These concentrations are compared to the air quality index (AQI) [27]. In general, low-PM tissues do not pose a significant risk of particulate exposure for the surgical team, but high-PM tissues pose a significant risk of exposure to operating room personnel even if they are away from the immediate vicinity of the source. Based on our estimates in Table 3, we can see that in high-PM procedures, even with integrated smoke evacuation, the AQI classification at 30 cm distance from the cutting point remains Very High. Very Low classifications are only achieved at distances of 1 m (or more) when using integrated evacuation, and at 2 m when using general-purpose surgical suction.

**Table 3:** Spherical model approximation for particle concentrations from various distances, for the tested tissues.

Extrapolated particulate exposures	Distance (cm)	High-PM μg/m3, AQI		Medium-PM μg/m3 AQI		Low-PM μg/m3 AQI	
**40 W, without any smoke removal**	30	1700	VH	500	VH	86	H
	50	360	VH	110	VH	19	L
	100	46	M	14	VL	2.3	VL
	200	5.6	VL	1.7	VL	0.29	VL
**40 W, with general purpose surgical suction (-50%)**	30	870	VH	260	VH	44	M
	50	190	VH	56	VH	9.7	VL
	100	24	L	7.1	VL	1.2	VL
	200	2.9	VL	0.88	VL	0.15	VL
**40 W, with integrated smoke evacuator (-88%)**	30	200	VH	60	H	10	VL
	50	44	M	13	VL	2.3	VL
	100	5.6	VL	1.7	VL	0.28	VL
	200	0.68	VL	0.21	VL	0.036	VL

A high-PM (liver), medium-PM (kidney, skeletal muscle) and low-PM (skin, subcutaneous fat, lung and brain). PM_2.5_ Air quality index (AQI) for one hour exposure. Very low (VL), Low (L), Medium (M), High (H), Very High (VH).

## Discussion

Our results show significant differences in the mass concentration and size distribution of the particles in SS from different porcine tissues. The tissues can be divided into three groups according to their particle production. Liver produces by far the highest number of particles. Renal tissues and skeletal muscle produce a medium mass of particulate matter, while subcutaneous fat, lung tissue, bronchus, cerebral gray and white matter, and skin produce significantly less particulate mass. Some of the tested tissues have large variations in the number of particles that they produce (Fig 4). These variations can be explained by the heterogeneity of the histologic structures within the tissue specimens, such as connective tissue, blood vessels and hematomas, which can result in significant differences in the composition of the smoke produced by the same specimen.

Our results differ from those of Hinz et al. [28]. While they observed similar particle distributions from the liver and the kidney, we noted a pronounced difference between these tissues. However, other research groups, such as Bruske-Hohlfeld et al. [29] and Pillinger et al. [26], have presented results that support our findings. Bruske-Hohlfeld et al. reported the largest particle concentrations from the surgery of hemangioma of liver, which is in line with our results, even though a liver hemangioma and a porcine liver differ from each other histologically.

According to Pillinger et al., the mean mass concentration of particle exposure for a surgeon was 137 μg/m^3^ when a smoke evacuation unit was not used [26]. This result is well in line with our estimate for the surgeon’s particle exposure using the spherical model. At operating distances, without a dedicated smoke evacuation system, high-PM and medium-PM tissues produce PM_2.5_ concentrations over 150 μg/m^3^. According to European Union air quality indices [28] this is unhealthy. Wang et al. [24] observed that a wall-installed smoke evacuation unit reduced the PM_2.5_ concentration by approximately half. Additionally, Pillinger et al. observed a particle mass reduction of 88% [26], when a suction device was integrated into the surgical blade. According to our results, is seems that with a smoke evacuation system, the surgeon is only exposed to unhealthy concentrations of particulate matter when operating on high-PM tissues, whereas general wall suction is adequate only for low-PM tissues.

In addition to smoke evacuators, the concentration of inhaled particles can be reduced with surgical masks. Nevertheless, there are vast differences in the filtering efficiencies of such masks, which can range from 13% to 99% [16]. In fact, it appears that only N95-respiratory protectors can be regarded as being more efficient than smoke evacuators in reducing surgical operating personnel’s exposure to particulates in SS [30]. According to the AQI classification [27], surgical theatre personnel can be exposed from very low to very high doses of PM during electrosurgery, so we suggest using a combination of masks and smoke evacuators for electrosurgery on high-PM tissues, depending on the filtering options and the tissue being operated on. Even though there have been no epidemiological studies that show an elevated lung cancer risk in OR personnel [31], PM exposure is associated with an increased risk of lung cancer, higher mortality [32] and a higher risk of persistent airway problems [33]. Even though electrosurgery is used only for a fraction of the time during a whole surgical procedure, some OR personnel, especially plastic surgeons, and urologic and general surgical practitioners do make extensive use of electrosurgery. Without adequate protection, this population may be at risk of long-term health problems related to PM exposure. Our results and the proposed PM class division could be used as a reference for any surgeon when selecting protective measures for an operation. For example, if the surgery only involves low-PM tissue surgery, the workplace safety requirements for surgical masks would be lower than for medium-PM and high-PM tissue operations. However, in order to validate these PM classes as a standard practice, more particle measurements with a larger sample sizes are needed, including further testing of the filtering efficiency of different types of surgical masks.

It must be acknowledged that there may be some limitations and contradictions in our study. Krones et al. studied the production of volatile compounds (VOC) in electrosurgery with porcine tissue samples and found that the VOC ratio between liver and fat was significantly less than our aerosol ratio between the liver and subcutaneous fat [34]. However, VOC and aerosol ratios may not be uniform. Another possible source of inaccuracy could be that our diathermy unit does not compensate for variations in the tissue impedance, but instead operates with a constant voltage. Tissue impedance compensation is used in some diathermy devices to produce more balanced cutting efficiency for different tissue types [35]. As the tested tissue types had different impedances, the results may have been different if the experiment had been done with an instrument with impedance compensation. On the other hand, our results do accurately reflect the relation between natural tissue impedance and smoke production, without any bias from such compensation features. We standardized the cuts, so we could directly measure the variations in smoke production from the heterogeneous samples without any significant variance in the cuts themselves.

Natural aerosol particle size distributions typically have multiple modes, e.g. the aerosol emission from a vehicle exhaust typically contains a nucleation particle mode (median particle diameter around 10 nm) and a soot particle mode (median particle diameter around 50 nm). Unfortunately, the ELPI size range is limited and particles smaller than 10 nm are only partly detected. The particle size distribution curves presented in Fig 3 indicate that there are two particle modes: the first around 10 nm and the second around 100 nm, but the curves are similar to those of ELPI measurements in vehicle exhaust emissions [36] [37].

## Conclusions

Our results indicate significant differences in particle production from different types of tissue during electrosurgery. The results suggest that the tissues can be divided into three groups according to their particle emissions: high-PM tissues (liver), medium-PM tissues (renal cortex, renal pelvis, muscle), and low-PM tissues (skin, cerebral gray matter, cerebral white matter, bronchus, subcutaneous fat). These classes can be related to surgery-specific PM doses. These results are of clinical importance for the protective measures used by surgeons and OR staff who employ electrosurgery extensively. We recommend smoke evacuation and particulate filtration masks, especially for high-PM and medium-PM tissue surgery. More studies on smoke production and mask efficiency are still needed in order to produce yet more accurate health hazard limits and practical recommendations for electrosurgical procedures.

## Supporting information

S1 Dataset Raw data from the ELPI. Consists the ELPI measurement data from the performed experiments.
(MAT)

S1 File Calculations and visualization for the particle size and number distributions. Including the results of each individual burning test. To execute this file, Matlab^®^ R2016a or later is required.
(M) Click here for additional data file.

S1 Table Tissue specific timing in the experiments. This file is required to execute the S1 File.
(MAT)
